# Analysis of Fungal and Bacterial Co-Infections in Mortality Cases among Hospitalized Patients with COVID-19 in Taipei, Taiwan

**DOI:** 10.3390/jof8010091

**Published:** 2022-01-17

**Authors:** De-En Lu, Shih-Han Hung, Ying-Shih Su, Wen-Sen Lee

**Affiliations:** 1 Department of Internal Medicine, Wan Fang Medical Center, Taipei Medical University, Taipei 116, Taiwan; louis2957@gmail.com; 2 Department of Otolaryngology, Wan Fang Medical Center, Taipei Medical University, Taipei 116, Taiwan; seedturtle@gmail.com; 3 Department of Otolaryngology, School of Medicine, College of Medicine, Taipei Medical University, Taipei 116, Taiwan; 4 Division of Infectious Diseases, Department of Internal Medicine, Wan Fang Medical Center, Taipei Medical University, Taipei 116, Taiwan; 109005@w.tmu.edu.tw; 5 Department of Internal Medicine, School of Medicine, College of Medicine, Taipei Medical University, Taipei 116, Taiwan

**Keywords:** fungal infections, bacterial infections, co-infections, COVID-19, mortality

## Abstract

Fungal or bacterial co-infections in patients with H1N1 influenza have already been reported in many studies. However, information on the risk factors, complications, and prognosis of mortality cases with coronavirus disease 2019 (COVID-19) are limited. We aimed to assess 36 mortality cases of 178 hospitalized patients among 339 patients confirmed to have had SARS-CoV-2 infections in a medical center in the Wenshan District of Taipei, Taiwan, between January 2020 and September 2021. Of these 36 mortality cases, 20 (60%) were men, 28 (77.7%) were aged >65 years, and the median age was 76 (54–99) years. Comorbidities such as hypertension, coronary artery disease, and chronic kidney disease were more likely to be found in the group with length of stay (LOS) > 7 d. In addition, the laboratory data indicating elevated creatinine-phosphate-kinase (CPK) (*p* < 0.001) and lactic acid dehydrogenase (LDH) (*p* = 0.05), and low albumin (*p* < 0.01) levels were significantly related to poor prognosis and mortality. The respiratory pathogens of early co-infections (LOS < 7 d) in the rapid progression to death group (*n* = 7 patients) were two bacteria (22.2%) and seven *Candida* species (77.8.7%). In contrast, pathogens of late co-infections (LOS > 7 d) (*n* = 27 patients) were 20 bacterial (54.1%), 16 *Candida* (43.2%), and only 1 *Aspergillus* (2.7%) species. In conclusion, the risk factors related to COVID-19 mortality in the Wenshan District of Taipei, Taiwan, were old age, comorbidities, and abnormal biomarkers such as low albumin level and elevated CPK and LDH levels. Bacterial co-infections are more common with Gram-negative pathogens. However, fungal co-infections are relatively more common with *Candida* spp. than *Aspergillus* in mortality cases of COVID-19.

## 1. Introduction

The novel coronavirus disease was first reported in Wuhan, China, in December 2019 (hence the name, COVID-19) and is caused by severe acute respiratory syndrome coronavirus 2 (SARS-CoV-2). COVID-19 has severely impacted the healthcare system globally. To date, more than 15,000 confirmed cases and 898 deaths have been reported in Taiwan. However, information concerning the clinical impact of co-infections with fungi or bacteria in COVID-19 mortality patients is still limited, and results vary in different areas of the world.

Co-infections with pulmonary aspergillosis in influenza (H1N1) patients are-well known, have been described in many studies, and may be related to air pollution [1,2,3,4,5,6]. However, co-infections between SARS-CoV-2 and other respiratory pathogens have become a serious concern in the treatment of patients with COVID-19 [2,4,7,8,9,10,11]. Many bacterial species, such as *Streptococcus pneumoniae*, *Mycoplasma pneumoniae*, *Legionella pneumophila*, *Staphylococcus aureus*, *Haemophilus influenzae*, *Klebsiella pneumoniae*, and *Pseudomonas aeruginosa*, and several viruses, such as the influenza virus, rhinovirus/enterovirus, non-SARS-CoV-2 coronavirus, respiratory syncytial virus, parainfluenza, and metapneumovirus, have been reported as possible co-pathogens among COVID-19 patients [4,7,8,9,10,11]. Rarely, co-fungal infections with COVID-19 have also been reported in the literature, and the reported pathogens include *Candida*, *Cryptococcus*, *Mucorales*, and *Aspergillus* spp. [2,4,9,12]. This retrospective study aimed to analyze the related risk factors of comorbidities, as well as common fungal or bacterial pathogens of co-infections in mortality cases of COVID-19 between January 2020 and September 2021 in Taipei, Taiwan.

## 2. Material and Methods

**Figure 1:**
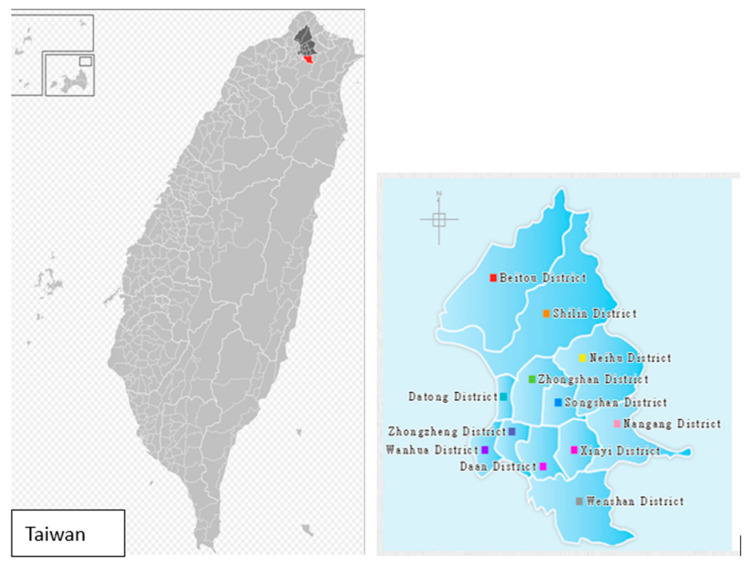
The Wenshan District (red color), located at the border of Taipei City and near the central mountain range (reference from Google Maps).

We collected and analyzed data on 36 mortality cases of SARS-CoV-2 infections between January 2020 and September 2021 from a medical center in the Wenshan District of Taipei, Taiwan, which were confirmed by reverse-transcription polymerase chain reaction (RT-PCR) (Figure 1). The patients were subdivided into two groups according to the length of the hospital stay (LOS): <7 d (*n* = 9) and >7 d (*n* = 27). Bacterial or fungal co-infection was defined as early infection (LOS < 7 d) and late infection (LOS > 7 d). We analyzed the demographic data, laboratory tests, radiographic findings, comorbidities, and microbiological results obtained from electronic resources and the clinical medical records of Wan Fang Medical Center.

Invasive pulmonary aspergillosis (IPA) was defined as concurrent pneumonia and a positive test for *Aspergillus* galactomannan (GM) antigen from serum, bronchoalveolar lavage fluid (BAL), and/or endobronchial secretion. Pneumonia was defined as described previously [4]. Diagnosis of COVID-19 and influenza was based on a throat swab positive for COVID-19 and an influenza test using one of the following polymerase chain reaction (PCR) analyses: for COVID-19, influenza A, influenza A (H1N1), influenza A (H3N2), or influenza B. Severe COVID-19 referred to any clinically severe SARS-CoV-2 infection making intensive monitoring and advanced supportive care necessary and requiring the affected patient to be admitted to an ICU. It is a notifiable disease in Taiwan and is therefore required by law to be reported to the Taiwan Centers for Disease Control. The identification of the microorganisms used matrix-assisted laser desorption ionization time-of-flight mass spectrometry (MALDI-TOF), and the susceptibility test used commercial Phoenix automated machine kits (BD company, Phoenix, AZ, USA).

## 3. Results

**Figure 2:**
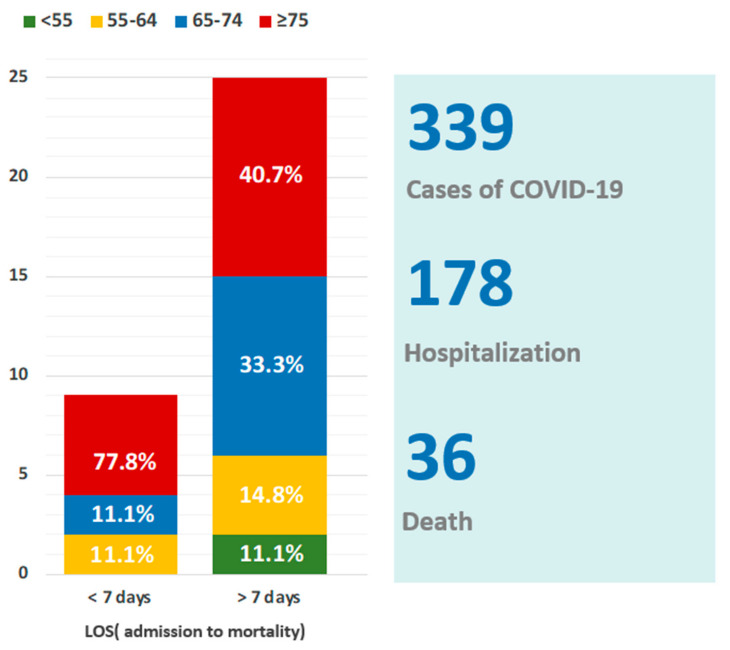
Epidemiology and age range of patients with COVID-19. COVID-19, Coronavirus disease 2019. LOS, length of stay (admission to mortality).

**Figure 3:**
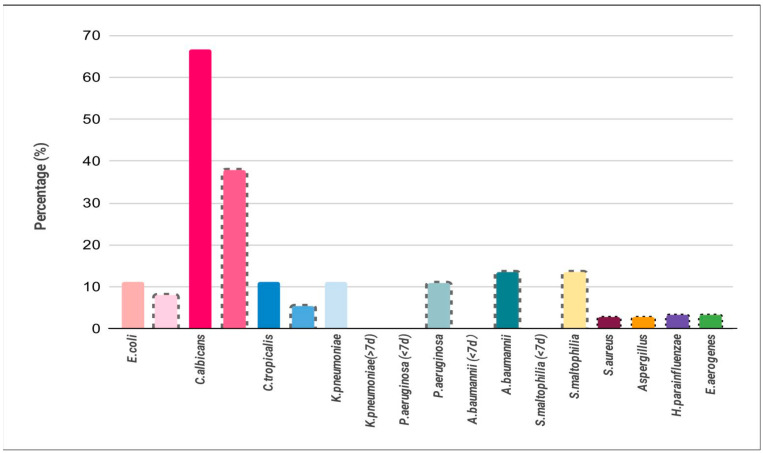
Distribution of respiratory pathogens in COVID-19 mortality cases. The biologic sample was aspirated from endo-tracheal aspirate. *C. albicans* was the most common co-infection. Solid line: <7 d length of stay (LOS); Dotted line: >7 d LOS.

There were 339 patients with COVID-19 infections noted during the study period, and 178 patients were admitted to the ward for management and treatment (Figure 2). Among these hospitalized patients, we assessed data on 36 mortality patients with SARS-CoV-2 infections who died during hospitalization. Of these, 20 (60%) were men, and 28 (77.7%) were aged >65 years, with a median age of 76 (54–99) years (Table 1). Comorbidities such as hypertension, coronary artery disease, and chronic kidney disease were more likely to be found in mortality patients and showed an increased proportion in the group with LOS > 7 d (Table 1). In addition, the laboratory data (biomarker) for patients with LOS (>7 d) showed significantly elevated creatinine-phosphate-kinase (CPK) (*p* < 0.001) and lactic acid dehydrogenase (LDH) (*p* = 0.05) and low albumin levels (*p* < 0.01) (Table 2). The pathogens of early co-infections (LOS < 7 d) in the rapid progression to death group (*n* = 7 patients) (Table 3 and Table 4 ) were two bacterial (22.2%) and seven *Candida* species (77.8.7%). Contrastingly, pathogens of late co-infections (LOS > 7 d) (*n* = 27 patients) were 20 bacterial (54.1%), 16 *Candida* (43.2%), and 1 *Aspergillus* (2.7%) species (Figure 3).

## 4. Discussion

While bacterial and viral co-infections at the time of SARS-CoV-2 diagnosis (community infections) seem to be rare in early hospitalization [1,2,3], co-infections commonly arise in the late period of hospitalization in patients with COVID-19 [1,2,3,4], with their frequency increasing with the severity of the disease. A detailed pathological discussion of the mechanisms underlying the susceptibility to healthcare-associated infections in these subjects is beyond the scope of this study. However, several factors, such as comorbidities (Table 4), immune-modulating therapies, widespread use of empirical antimicrobial drugs, and SARS-CoV-2-related pathological derangements of the immune system and epithelial barriers are likely to play a role [1,2,3,4,12,13]. Notably, several reports suggest that patients with COVID-19 could be at a higher risk of developing secondary infections when compared to patients with either bacterial [4] or viral pneumonia or invasive pulmonary aspergillosis [4,5,6,7,8]. Although classical invasive pulmonary aspergillosis (IPA) leads to typical pulmonary cavitary lesions in severely immunocompromised patients, there were higher chances of IPA in patients with clinically severe COVID-19 or severe influenza, who were reported to exhibit various types of pulmonary lesions [4,5,6,7,8,9]. IPA can also occur in patients with modified immune impaired disorders or in critically ill patients who are potentially vulnerable to IPA [10,11,12,13,14]. The presence of IPA in 17% of patients suffering from clinically severe COVID-19 or severe influenza in Taiwan [2,7,8,9,10,15,16,17,18,19,20,21] seemed to be related to a prior high-level ambience. In our study, the IPA of mortality cases of COVID-19 was very low. This result may be due to the relatively good air quality of the Wenshan District of Tapei City, because this district is near the central mountain range of Taiwan (Figure 1) [5,6,11].

Interestingly, this increased incidence of superinfections did not seem to be driven by pre-existing risk factors predisposing patients to healthcare-associated infections (e.g., diabetes and immunosuppression), suggesting that SARS-CoV-2 infection could be associated with a heightened risk of infectious complications [14,15,16,17,18].

The microorganisms shown to be most commonly involved in co-infections or secondary infections are bacteria and *Candida* spp. [18,19,20], while *Aspergillus* is less frequently implicated, which is consistent with our study [16,17,18,19]. In a minority of patients, an increased incidence of invasive candidiasis in patients with COVID-19 even after adjustment for other common risk factors associated with *Candida* infection was observed [16,17,18,19,20,21]. This observation led to speculation that SARS-CoV-2 infection could, by itself, be associated with a heightened risk of candidiasis through different pathogenic mechanisms. SARS-CoV-2 can infect enterocytes, leading to decreased integrity of the intestinal barrier [18,19,20,21,22]. Increased concentrations of plasma markers of microbial translocation have been reported in infected patients, and the microbiota of COVID-19 patients were found to be skewed toward an increased presence of *Candida* spp. [17,18,19,20,21]. These findings suggest that this population is at an increased risk of *Candida* translocation and subsequent candidemia. Furthermore, the pathology of severe SARS-CoV-2 infection involves profound dysregulation of the immune system. Based on the cellular activation profile and cytokine secretion, different host immune phenotypes can be distinguished during COVID-19 [23,24]. These immunotypes range from “hyper-inflamed”, with increased T-lymphocytes and myeloid cell expression of activation markers, to “exhausted”, in which immune cells of the affected patient are enriched in immune-exhaustion markers and fail to respond to toll-like receptor engagement and pathogen exposure ex vivo [24,25]. Among these clinical and immunological conditions, response to commensal pathogens, such as *Candida* spp., could be impaired, and invasive candidiasis could develop more frequently. Noteworthily, a recent study reported a decreased response to ex vivo stimulation with *Candida* lysate in whole-blood cells derived from COVID-19-infected patients. This impairment was not observed when the same cells were stimulated with either bacterial antigens or *Aspergillus fumigatus* lysate, suggesting a possible pathogen-specific weakening of the immune response [15,16,17,18,19,20,21]. Although interesting, these observations are mostly derived from studies with small sample sizes and those enrolling patients with different degrees of disease severity, two factors that impede the generalization of the described mechanisms [15,16].

In addition, co-infection between SARS-CoV-2 and other respiratory pathogens has become a serious concern in the treatment of patients with COVID-19 [4,5,6,7,8,9,25]. Many bacteria (e.g., *S. pneumoniae*, *M. pneumoniae*, *L. pneumophila*, *S. aureus*, *H. influenzae*, *K. pneumoniae*, *P. aeruginosa*) and viruses (e.g., influenza virus, rhinovirus/enterovirus, non-SARS-CoV-2 coronavirus, respiratory syncytial virus, parainfluenza, metapneumovirus) have been reported as possible co-pathogens among COVID-19 patients [4,5,6,7,8,9,10,11]. Increasing co-fungal infections with COVID-19 have also been reported recently, and the pathogens include *Candida*, *Cryptococcus*, *Mucorales*, and *Aspergillus* spp. [2,4,8,9,12,15,16,19].

Among these possible co-pathogens in COVID-19 patients, attention should be focused on *Aspergillus*, because IPA is difficult to diagnose and can be associated with high morbidity and mortality rates [12,13,14,15]. Co-infection with IPA in severe influenza patients has recently been reported in the Netherlands, Belgium, Taiwan, and China [7,8,11]. Based on the experience of severe influenza-associated IPA, IPA might comprise up to 17–29% of severe influenza patients and contribute to a high mortality rate of up to 67% [12,20,21]. The IPA following respiratory viral infections is not limited to the influenza virus but may also include the respiratory syncytial virus or parainfluenza virus, SARS, human herpesvirus 6, and adenovirus [7,8,18,19,20]. However, existing studies and knowledge on the association of COVID-19 with pulmonary aspergillosis are limited. Therefore, we conducted a comprehensive review of the literature reporting co-pulmonary aspergillosis in patients with COVID-19 to provide updated information.

A proposed pathogenetic mechanism involving the role of interleukin (IL)-10 and IL-6 can be considered when analyzing the association between aspergillosis and COVID-19. IL-10 plays a key role in the regulation of cellular immune responses and is involved in a multitude of inflammatory diseases [4,25]. In a rat model, aspergillosis was significantly associated with an increase in IL-10 levels, correlating to an increased T helper 2 (Th2) cell response and a decreased Th1 response, resulting in a downregulation of macrophage activity and an increase in host susceptibility to *Aspergillus* infection [4,12,24,25].

The clinical significance of our study lies in showing that critically ill COVID-19 patients received a remdesivir, steroid, and monoclonal antibody combination therapy leading to the immunocompromised state; therefore, the *Candida* infection was seen in the early (<7 d) and late (>7 d) co-infections, respectively. Additionally, Gram-negative bacteria were more predominant than Gram-positive bacteria (e.g., *S. aureus*) [26]. Overall, the findings of the present study provide important information. First, in addition to common bacteria and viruses, *Candida* spp. can cause co-infections in patients with COVID-19, especially in severe or critical cases. Most importantly, the survival outcomes of these patients were poor. Acute respiratory distress syndrome (ARDS) requiring mechanical ventilator (MV) support was a common complication among these patients, and the overall mortality rate was high. Second, the conventional risk factors for invasive aspergillosis were not common among these populations. Therefore, clinicians should be aware of the possible occurrence of co-infection with invasive candidiasis in COVID-19 patients. Fungal culture and galactomannan tests, especially from respiratory specimens, could help in early diagnosis. Third, *Candida* spp. was the most common cause of co-infection in COVID-19 patients, followed by Gram-negative bacteria and *Aspergillus* spp. Although fluconazole and echinocandin are the recommended and most commonly used antifungal agents, breakthrough fungal infections caused by azole-resistant pathogens are also possible.

## 5. Conclusions

Old age, comorbidities, and abnormal biomarkers (low albumin level and elevated creatinine-phosphate-kinase (CPK) and lactic acid dehydrogenase (LDH) levels) are commonly seen in the initial presentation of mortality cases. Co-infections with *Candida* spp. and bacteria are also crucial factors in these patients. Clinicians should be aware that co-infections with severe COVID-19 viral pneumonia seem to be associated with invasive fungal and bacterial infections and correlated with numerous risk factors, such as the use of immunosuppressants and corticosteroids, as well as the duration of mechanical ventilation and ICU stay. These findings led us to consider antimicrobial prophylaxis in selected high-risk patients.

## Figures and Tables

**Table 1:** Demographics, baseline characteristics, and treatment of the patients with COVID-19 infections.

Length of Admission to Mortality (Days)	<7 Days	>7 Days	Overall	*p* Value
	*n* = 9	*n* = 27	*n*= 36	
Age (year)				
<55	0	3	3	
55–64	1	4	5	
≥65	8	20	28	<0.05
Sex				0.334
Female	2	14	16	0.343
Male	7	13	20	0.382
Active smoker	2	7	9	0.238
Comorbidities				
Hypertension	7	12	20	0.342
Diabetes mellitus	5	10	15	0.214
Coronary artery disease	5	17	22	0.183
Chronic kidney disease	5	17	22	0.188
Dyslipidemia	4	5	9	0.215
Hepatitis B	1	10	11	0.234
COPD/Asthma	2	6	8	0.328
Malignancy	0	7	7	0.181
Anti-COVID treatment				
Remdesivir	5	21	26	0.214
Tocilizumab	5	13	18	0.292
Dexamethasone	8	25	33	0.391

**Table 2:** Vital signs, cycle threshold (Ct) values, and biomarkers by patients’ groups.

Length of Admission to Mortality (Days)	<7 Days	>7 Days	Overall	*p* Value
	*n* = 9	*n* = 27	*n* = 36	
Age	81 ± 9.4	73.7 ± 13.6	75.6 ± 13	0.046
Body mass index	24.2 ± 4.9	24.4 ± 3.3	24.4 ± 3.7	0.427
Respiratory rate	20.4 ± 4.7	22.4 ± 4.2	21.6 ± 4.3	0.173
Heart rate	111.5 ± 22.6	95.8 ± 23.2	99.7 ± 23.8	0.047
Body temperature	37.3 ± 1.5	37.5 ± 1.1	37.5 ± 1.2	0.38
Cycling threshold	37.3 ± 1.5	23.5 ± 7.9	22.4 ± 6.5	0.182
WBC (10^3^/µL)	8 ± 3.6	10.1 ± 8.2	9.6 ± 7.3	0.236
Neutrophil (%)	86.8 ± 10.1	82.23 ± 10	84.1 ± 10	0.183
Lymphocyte (%)	7.3 ± 6.4	8.2 ± 5.7	8 ± 5.8	0.346
Monocyte (%)	5.1 ± 4	6.5 ± 4.5	6.1 ± 4.4	0.197
Albumin (g/dL)	3.3 ± 0.5	2.9 ± 0.5	3 ± 0.5	0.01
LDH (U/L)	554 ± 175	423 ± 201.2	451 ± 201.3	0.05
CPK (U/L)	758.2 ± 936	141.9 ± 138.5	300.4 ± 543.1	<0.001
D-dimer (mg/L)	4 ± 3.8	7 ± 7.3	6.3 ± 6.7	0.06
Fibrinogen (mg/dL)	429.1 ± 201.1	547.3 ± 244.4	519.5 ± 237.6	0.093
Platelet (10^3^/µL)	149 ± 47.6	190.5 ± 116.5	180.1 ± 104.5	0.072
Ferritin (ng/mL)	2053.1 ± 1106.5	3135 ± 5754.7	2864.5 ± 5010.6	0.18
CRP (mg/dL)	10.8 ± 6.7	13.4 ± 10.3	12.7 ± 9.5	0.19
ESR (mm/1 h)	55.4 ± 24.9	48.7 ± 35.9	50.4 ± 33.2	0.27

**Table 3:** The pathogens of co-infections of COVID-19 mortality cases.

Length of Admission to Mortality	<7 Days	>7 Days	Overall
Patient	*n* = 9	*n* = 27	*n*= 36
Sputum culture			
*Aspergillus*	0	1 (2.7%)	1 (2.2%)
*Candida albicans*	6 (66.7%)	14 (37.8%)	20 (43.4%)
*Candida tropicalis*	1 (11.1%)	2 (5.4%)	3 (6.5%)
*Escherichia coli*	1 (11.1%)	3 (8.1%)	4 (8.7%)
*Pseudomonas aeruginosa*	0	4 (10.8%)	4 (8.7%)
*Acinetobacter baumannii*	0	5 (13.5%)	5 (10.9%)
*Klebsiella pneumoniae*	1 (11.1%)	0	1 (2.2%)
*Stenotrophomonas maltophilia*	0	5 (13.5%)	5 (10.9%)
*Staphylococcus aureus*	0	1 (2.7%)	1 (2.2%)
*H. parainfluenzae*	0	1 (2.7%)	1 (2.2%)
*Enterobacter aerogenes*	0	1 (2.7%)	1 (2.2%)
Total	9	37	46

**Table 4:** Multivariate logistic regression analysis among hospitalized COVID-19 patients and mortality cases (142 survived vs. 36 mortality).

	Odds Ratio	95% Confidence Interval		*p*-Value
Age		lower	upper	
Age < 55	16.93	1.45	198.08	<0.02
Age 55–64	25.4	2.48	259.90	0.006
Age > 65	73.37	19.81	271.80	<0.0001
Chronic kidney disease	18.14	6.38	51.57	<0.0001
Diabetes mellitus type 2	33.86	8.57	133.77	<0.0001
Mortalities (coinfections vs. non-coinfections)	67.63	21.04	218.04	<0.0001

*p* < 0.05, insignificant variables excluded from the multivariate logistic regression.
